# Condom migration after introduction of pre-exposure prophylaxis among HIV-uninfected adolescents in South Africa: A cohort analysis

**DOI:** 10.4102/sajhivmed.v18i1.712

**Published:** 2017-09-21

**Authors:** Lennart P. Maljaars, Katherine Gill, Philip J. Smith, Glenda E. Gray, Janan J. Dietrich, Gabriela B. Gomez, Linda-Gail Bekker

**Affiliations:** 1 Desmond Tutu HIV Centre, University of Cape Town, South Africa; 2 Amsterdam Institute of Global Health and Development, the Netherlands; 3 South African Medical Research Council, South Africa; 4 Perinatal HIV Research Unit, University of Witwatersrand, South Africa; 5 Department of Global Health, Academic Medical Centre, the Netherlands

## Abstract

**Background:**

Approximately 3 million adolescents and young adults (AYA), between the ages of 15 years and 24 years, are living with HIV in sub-Saharan Africa. The use of pre-exposure prophylaxis (PrEP) may be a promising HIV prevention tool to bridge the high-risk years of AYA between sexual debut and adulthood.

**Objectives:**

Concerns have been raised that the use of PrEP could lead to an increase in sexual risk behaviour and sexually transmitted infections in general and less condom use in particular among adolescents.

**Methods:**

This study assesses condom use among South African adolescents enrolled on a demonstration PrEP study, called Pluspills, being conducted in Cape Town and Soweto. A questionnaire on sexual risk behaviour was administered at baseline and after 4, 8 and 12 weeks. Three different questions on condom use were asked at each visit. Unless all answers indicated condom use at all times, a participant was scored ‘at risk’. McNemar’s tests and a Cochran’s Q test were used to investigate changes in condom use over time.

**Results:**

We interviewed 148 adolescents (66% female) at baseline. Eighty-nine participants completed all visits. In this group, an increase in condom use was observed over the period of 12 weeks. Most participants who reported behavioural changes mentioned an increase in condom use.

**Conclusion:**

There was no sign of sexual risk compensation in the 12 weeks of the study. Observed increase in condom use can be explained by an increased awareness of personal HIV risk or by social desirability or recall biases. In future research, additional data including other biomarkers of unprotected sex and longer follow-up time would be useful to help understand the relationship between PrEP use, sexual risk perception and consequent behaviours, especially in adolescents.

## Introduction

### Background

HIV incidence reduction has plateaued in the recent years with some population sub-groups still being affected disproportionately.^1^ One of these key populations is adolescents and young adults (AYA) with approximately 2.9 million living with HIV in sub-Saharan Africa between the ages of 15 years and 24 years.^2^ Research shows that, because of a number of developmental, psychological, social and structural transitions that converge in this period of the lifespan, AYA are at increased risk of HIV acquisition.^3^ Moreover, a growing body of neurobiological research and imaging studies suggest that adolescents may be prone to engage in risk behaviours, including sexual risk taking and substance abuse, because of developmental changes.^4^ Risk factors for HIV acquisition seen in South African youth are the early age of sexual debut, a high number of sexual partners and inconsistent use of condoms.^5,6^

### Objectives

At the end of 2015, the South African Medicines Control Council approved a fixed-dose combination of tenofovir disoproxil fumarate and emtricitabine for pre-exposure prophylaxis (PrEP) of HIV, a new prevention tool in the fight against HIV.^7^ Previous research showed the benefits of PrEP if it is prioritised to high-risk populations, is adhered to and does not lead to an increase in risk behaviour.^8,9,10^ Therefore, the use of PrEP may be beneficial in preventing HIV infection during the high-risk years of youth, as PrEP could offer a time-limited strategy to bridge the developmental period in at-risk adolescents from sexual debut to adulthood. However, there is a concern that its use may result in risk compensation,^11^ that is, PrEP use might inhibit the uptake of safer behaviours by reducing people’s perception of their risk of infection.^12^

### Trends

In the past, the potential introduction of other HIV prevention technologies, such as vaccines, barrier methods,^13^ antiretroviral treatment for prevention, male circumcision^14^ and vaginal microbicides,^15,16^ raised the same public health concern. Yet, previous research has not found evidence for sexual risk compensation in adults using PrEP^17,18,19,20,21,22^ or post-exposure prophylaxis (PEP).^23^

### Contribution to the field

To date, there has been no research into sexual risk compensation among adolescents following the introduction of PrEP. With the ongoing roll-out of PrEP programmes and an upcoming United Nations Children’s Fund (UNICEF) funded project for PrEP implementation for adolescents in South Africa, this research question has become urgent. In particular there is potential concern that condom use may decrease in the short-term as a result of being substituted by another prevention intervention, also known as ‘condom migration’.^15^ In this article, we aim to explore short-term changes in condom use among HIV-uninfected adolescents using PrEP in South Africa.

## Research design

### Setting

This analysis was part of the Pluspills study^24,25^ which evaluates the use and effectiveness of PrEP among HIV-uninfected adolescents in South Africa. The Pluspills study is an open-label, single-arm cohort study to evaluate patterns of PrEP use and patterns of condom use among adolescents aged 15 years to 19 years.

### Design

The aim was to evaluate the change in condom use (condom migration) among participants starting PrEP for a period of 12 weeks. All participants received PrEP (FTC/TDF) as part of a combination prevention package. This package consisted of an individualised adherence plan for PrEP, risk reduction counselling, free condoms and condom counselling, screening and treatment for sexually transmitted infections (STIs) and counselling and referral for other HIV prevention interventions (e.g. PEP, voluntary male circumcision). Sampling in the study was stratified by gender, because women are more vulnerable to HIV acquisition in this setting. Community engagement via the adolescent community advisory board (CAB) and a consent education discussion group prior to screening was done at both sites. The discussion groups enabled the study team to explain the prevention package, including PrEP, to participants and enabled participants to engage with the study team. After these meetings, participants were invited to enrol in the Pluspills study. The following inclusion criteria had to be met: participants must be aged between 15 years and 19 years old, willing to provide assent or consent, have a guardian willing to provide consent, able to provide adequate locator information, HIV-uninfected, sexually active, have negative pregnancy test, effective method of contraception, no intention to relocate, no activities that require long absences from the area and willing to undergo all study-required procedures. At enrolment, participants were asked to initiate PrEP as part of a comprehensive prevention package for at least three months. Thereafter individuals were allowed to opt out of PrEP use should they wish to, whilst remaining in the study. The choice to opt out was posed at each three-monthly visit. Participants were counselled at every visit as part of the combination prevention package. Counsellors and clinicians offered participants condoms at each visit. Condoms were also available in the bathrooms of each clinic.

### Procedure

A behavioural questionnaire was administered at baseline and at each subsequent visit. The questionnaire aimed to collect information on sexual behaviour, risk taking and changes over time.

### Measurements and coding

The three questions on condom use (consistency of condom use, condom use during last sexual act and frequency of condom use) were coded as ‘at risk’ (=1) and ‘not at risk’ (=0). Only an explicit confirmation of condom use on all three questions was scored as ‘not at risk’. Inconsistent condom use was defined as any uncertainty in the self-report on condom use. If a participant scored 1 on any of the three questions she/he was considered ‘at risk’. Participants were also asked if and how they changed their sexual behaviour between the different time points. Self-reported change was used to assess participants’ perception of sexual risk. Participants were screened for STIs (Herpes Simplex Virus-2, *Chlamydia trachomatis* and *Neisseria gonorrhoeae*) at baseline and week 12.

### Statistical analyses

Baseline condom use data were compared with data points after 4, 8 and 12 weeks and these were also compared with each other. McNemar’s tests and a Cochran’s Q test were used to look for change in condom use over time. In particular, the McNemar’s test was used to see if change in condom use (=condom migration) was random or because of the intervention. Data were analysed using STATA 13.0.

#### Ethical consideration

This study was approved by the Division of AIDS (DAIDS), Independent Ethics Committees, and South Africa’s Medicines Control Council prior to implementation. This study was conducted according to the protocol as well as the International Conference on Harmonisation of Technical Requirements for Registration of Pharmaceuticals for Human Use (ICH) and South African Good Clinical Practices.

## Results

### Demographic information

Eighty-nine participants completed all subsequent visits (see Tables 1 and 2): 41 participants at the Desmond Tutu HIV Foundation Youth Centre in Masiphumelele, Cape Town and 48 participants at the Perinatal HIV Research Unit in Soweto, Johannesburg. Of the participants who completed all visits, 58 (65%) were females, as per design. All participants were of black ethnicity and lived in the townships of Masiphumelele or Soweto. At baseline 75% of the participants attended school and 11% attended a tertiary education institute. Eighty percent of the sample lived with, at least one of their parents; the other 20% lived with their grandparents, other family or a guardian. Two-thirds of the sample reported inconsistent condom use at baseline. Female participants reported more inconsistent condom usage than male participants at baseline. The STI numbers were high, with 37% of the participants having an STI at baseline. STIs were more prevalent in female participants than in male participants, respectively 47% versus 16%.

**TABLE 1:** Baseline characteristics.

Characteristic	Female (*n* = 58)		Male (*n* = 31)		All (*n* = 89)	
	*n*	%	*n*	%	*n*	%
**Location**						
Cape Town	26	44.8	15	48.4	41	46.1
Soweto	32	55.2	16	51.6	48	53.9
**School attendance**						
Yes	43	74.1	24	77.4	67	75.3
**Highest grade completed**						
Senior phase (grade 7–9)	17	29.3	13	41.9	30	33.7
FET band (grade 10–12)	41	70.1	18	58.1	59	66.3
**Attending tertiary education institute**						
Yes	6	10.3	4	12.9	10	11.2
**Household**						
Mother and/or father	47	81.0	24	77.4	71	79.8
Grandmother and/or -father	4	6.9	2	6.5	6	6.7
Other	7	12.1	5	16.1	12	13.5
Inconsistent condom use	44	75.9	17	54.8	61	68.5
Condom use (last sexual act)	39	67.2	24	77.42	63	70.8
Sexually transmitted infections	27	46.6	5	16.1	32	36.0

FET, further education and training.

**TABLE 2:** Age and average grade of participants.

Characteristic	Female (*n* = 58)				Male (*n* = 31)				All (*n* = 89)			
	Median	IQR	Mean	SD	Median	IQR	Mean	SD	Median	IQR	Mean	SD
Age in years	18.0	16.0–19.0	17.41	1.43	18.0	17.0–18.0	17.58	0.99	18.0	17.0–18.0	17.47	1.29
Average grade	10.0	9.0–12.0	10.29	1.31	10.0	9.0–11.0	10.00	1.32	10.0	9.0–11.0	10.19	1.31

*n*, number; IQR, interquartile range; SD, standard deviation.

### Follow-up sexually transmitted infections

In the follow-up, data regarding STI screening were only available for 81 out of the 89 participants. Of the participants, 30% had an STI (Table 3), and 19% had an STI at both time points. At week 12, five participants had an STI despite reporting consistent condom use on all intermediate time points.

**TABLE 3:** Sexually transmitted infections.

Characteristics	STI rates (week 12)					
	Yes		No		All (*n* = 81)	
	*n*	%	*n*	%	*n*	%
**Gender**						
Male	5	6.2	19	23.5	24	29.6
Female	19	23.5	35	43.2	57	70.4
**Sexual transmitted infection (baseline)**						
Yes	15	18.5	15	18.5	30	37.0
No	9	11.1	42	51.9	51	63.0
**Consistent condom use (week 4–12)**						
At risk	19	23.5	46	56.8	65	80.2
Not at risk	5	6.2	11	13.6	16	19.8

*n*, number; STI, sexually transmitted infection.

### Condom migration

**FIGURE 1:**
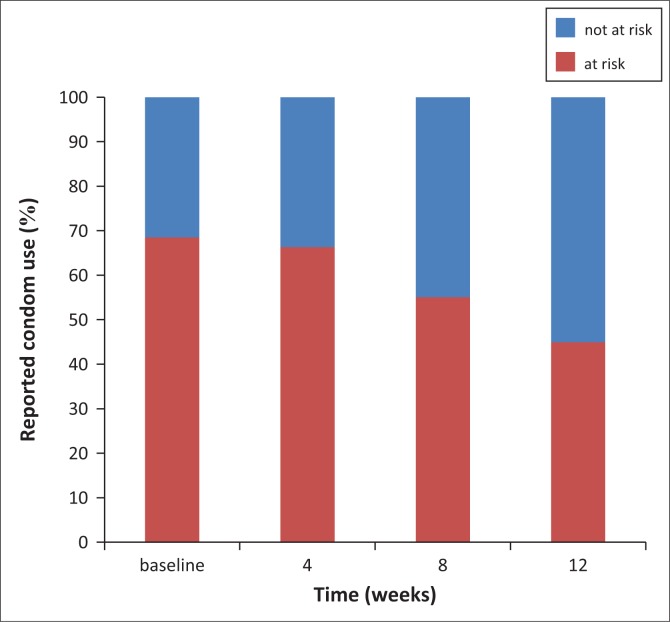
Condom migration (*N* = 89).

McNemar’s tests showed an increase in condom use between baseline and week 8, baseline and week 12, and week 4 and week 12 (see Table 4). The Cochran’s Q test with all data points also showed an increase in condom use over time (see Table 4). Approximately 28% of the participants reported a change in sexual behaviour between the different visits. Of them, 87% (81% – 92%) reported using condoms more often than before. During the follow-up period, fewer participants were classified ‘at risk’ because of more consistent condom use reported over time (see Figure 1).

**TABLE 4:** Change in condom use.

Time point	*p*
**McNemar’s test**	
Baseline & Week 4	0.6698
Baseline & Week 8	0.0285*
Baseline & Week 12	0.0008**
Week 4 & Week 8	0.0330*
Week 4 & Week 12	0.0006**
Week 8 & Week 12	0.106
**Cochran’s Q test**	
Baseline until Week 12	0.0002**

* *p* < 0.05

** *p* < 0.01.

## Discussion

### Outline of the results

We did not see a reduction in condom use over this relatively short period of time in a South African adolescent population commencing PrEP. Reported condom use was similar to previous research of condom use patterns in adolescents in South Africa.^5,26^ What is more is that in this relatively small sample we observed an increase in self-reported condom use. We can hypothesise multiple explanations for this finding. Firstly, this reporting is valid and may be because of the benefits of repeated risk reduction counselling, provision of condoms, enhanced awareness of potential risk because of provision of PrEP and more partner support because of enrolment in a study. Secondly, this finding can be false reporting as a result of social desirability or recall biases in self-reported condom use. Whilst ongoing surveillance of sexual risk compensation is warranted in PrEP implementation, these data are at least encouraging.

### Limitations

A total of 148 participants completed the baseline visit, but only 89 participants completed all three subsequent visits. Whilst a comparison of demographic baseline data and baseline reported sexual behaviour between participants who attended their visits and the ones who missed a visit showed no difference, the analysis was carried out only in those who attended all three survey time points. This limited the number of participants in the study. Secondly, a limitation of the study was that condom use was based on self-report which can be influenced by social desirability or recall biases. Using other biomarkers of unprotected sex may be a more reliable measure of current risk behaviour.^27^ STI rates can be a surrogate to validate self-reported condom use. Despite self-reported consistency in condom use, five participants had an STI after 12 weeks, perhaps raising some questions around the validity of consistent condom use.

## Conclusion and recommendations

To our knowledge, this is the first study of condom migration among adolescents on PrEP. Three months is a relatively short period, although this is the time that all adolescents were expected to consistently be using daily PrEP in Pluspills. Whilst more research is needed to understand the relationship between PrEP use, sexual risk behaviour, perception of risk and consequent behaviours, especially among adolescents, these preliminary and limited results are encouraging. Whilst PrEP may well be a very important intervention prevention addition to the tool box for adolescents, it is also understood that it should always be administered as part of a broader package that also includes risk reduction counselling, condom provision, contraception and STI screening as part of adolescent-friendly services.

## References

[1] UNAIDS Prevention gap report [homepage on the Internet]. Geneva; 2016 [cited 2016 Oct 10]. Available from: http://www.unaids.org/sites/default/files/media_asset/2016-prevention-gap-report_en.pdf

[2] UNAIDS The GAP report [homepage on the Internet]. Geneva; 2014 [cited 2016 Jul 14]. Available from: http://files.unaids.org/en/media/unaids/contentassets/documents/unaidspublication/2014/UNAIDS_Gap_report_en.pdf

[3] Bekker LG, Johnson L, Wallace M, Hosek S Building our youth for the future. J Int AIDS Soc. 2015;18:1–7. https://doi.org/10.7448/IAS.18.2.20027 10.7448/IAS.18.2.20027 PMC4344540 25724512

[4] Galvan A, Hare T, Voss H, Glover G, Casey BJ Risk-taking and the adolescent brain: Who is at risk? Dev Sci. 2007;10:F8–14. https://doi.org/10.1111/j.1467-7687.2006.00579.x 17286837 10.1111/j.1467-7687.2006.00579.x

[5] Pettifor AE, Rees HV, Kleinschmidt I, et al Young people’s sexual health in South Africa: HIV prevalence and sexual behaviors from a nationally representative household survey. AIDS. 2005;19(14):1525–1534. https://doi.org/10.1097/01.aids.0000183129.16830.06 16135907 10.1097/01.aids.0000183129.16830.06

[6] Eaton L, Flisher AJ, Aaro LE Unsafe sexual behaviour in South African youth. Soc Sci Med. 2003;56(1):149–165. https://doi.org/10.1016/S0277-9536(02)00017-5 12435558 10.1016/s0277-9536(02)00017-5

[7] Medicines Control Council Press release: Medicines Control Council approves fixed-dose combination of tenofovir disoproxyl fumarate and emtricitabine for pre-exposure prophylaxis of HIV [homepage on the Internet]. Pretoria; 2015 [cited 2016 Oct 31]. Available from: http://www.mccza.com/documents/2e4b3a5310.11_Media_release_ARV_FDC_PrEP_Nov15_v1.pdf

[8] Cremin I, Alsallaq R, Dybul M, Piot P, Garnett G, Hallett TB The new role of antiretrovirals in combination HIV prevention: A mathematical modelling analysis. AIDS. 2013;27(3):447–458. https://doi.org/10.1097/QAD.0b013e32835ca2dd 23296196 10.1097/QAD.0b013e32835ca2dd

[9] Gomez GB, Borquez A, Case KK, Wheelock A, Vassall A, Hankins C The cost and impact of scaling up pre-exposure prophylaxis for HIV prevention: A systematic review of cost-effectiveness modelling studies. PLoS Med. 2013;10(3):e1001401 https://doi.org/10.1371/journal.pmed.1001401 23554579 10.1371/journal.pmed.1001401 PMC3595225

[10] Pretorius C, Stover J, Bollinger L, Bacaër N, Williams B Evaluating the cost-effectiveness of pre-exposure prophylaxis (PrEP) and its impact on HIV-1 transmission in South Africa. PLoS One. 2010;5(11):e13646 https://doi.org/10.1371/journal.pone.0013646 21079767 10.1371/journal.pone.0013646 PMC2974638

[11] Alaei K, Paynter CA, Juan S-C, Alaei A Using PrEP, losing condoms? PrEP promotion may undermine safe sex. AIDS [serial online]. 2016 [cited 2016 Oct 31]. Available from: http://www.ncbi.nlm.nih.gov/pubmed/27662553 10.1097/QAD.0000000000001262 27824624

[12] Cassell MM, Halperin D, Shelton J, Stanton D Risk compensation: The Achilles’ heel of innovations in HIV prevention? BMJ. 2006;332(7541):605–607. https://doi.org/10.1136/bmj.332.7541.605 16528088 10.1136/bmj.332.7541.605 PMC1397752

[13] Posner SF, Van Der Straten A, Kang MS, Padian N, Chipato T Introducing diaphragms into the mix: What happens to male condom use patterns? AIDS Behav. 2005;9(4):443–449. https://doi.org/10.1007/s10461-005-9016-z 16235134 10.1007/s10461-005-9016-z

[14] Westercamp N, Agot K, Jaoko W, Bailey RC Risk compensation following male circumcision: Results from a two-year prospective cohort study of recently circumcised and uncircumcised men in Nyanza Province, Kenya. AIDS Behav. 2014;18(9):1764–1775. https://doi.org/10.1007/s10461-014-0846-4 25047688 10.1007/s10461-014-0846-4

[15] McMahon JM, Morrow KM, Weeks M, Morrison-Beedy D, Coyle A Potential impact of vaginal microbicides on HIV risk among women with primary heterosexual partners. J Assoc Nurses AIDS Care. 2011;22(1):9–16. https://doi.org/10.1016/j.jana.2010.05.001 21211700 10.1016/j.jana.2010.05.001 PMC3050650

[16] Foss AM, Vickerman PT, Heise L, Watts CH Shifts in condom use following microbicide introduction: Should we be concerned? AIDS. 2003;17(8):1227–1237. https://doi.org/10.1097/00002030-200305230-00015 12819525 10.1097/00002030-200305230-00015

[17] Carlo Hojilla J, Koester KA, Cohen SE, et al Sexual behavior, risk compensation, and HIV prevention strategies among participants in the San Francisco PrEP demonstration project: A qualitative analysis of counseling notes. AIDS Behav. 2016;20(7):1461–1469. https://doi.org/10.1007/s10461-015-1055-5 25835463 10.1007/s10461-015-1055-5 PMC4592687

[18] Grant RM, Anderson PL, McMahan V, et al Uptake of pre-exposure prophylaxis, sexual practices, and HIV incidence in men and transgender women who have sex with men: A cohort study. Lancet Infect Dis. 2014;14(9):820–829. https://doi.org/10.1016/S1473-3099(14)70847-3 25065857 10.1016/S1473-3099(14)70847-3 PMC6107918

[19] Guest G, Shattuck D, Johnson L, et al Changes in sexual risk behavior among participants in a PrEP HIV prevention trial. Sex Transm Dis [serial online]. 2008 [cited 2016 Dec 08];35(12):1002–8. Available from: https://www.ncbi.nlm.nih.gov/pubmed/19051397 19051397

[20] Marcus JL, Glidden DV, Mayer KH, et al No evidence of sexual risk compensation in the iPrEx trial of daily oral HIV preexposure prophylaxis. PLoS One. 2013;8(12):e81997 https://doi.org/10.1371/journal.pone.0081997 24367497 10.1371/journal.pone.0081997 PMC3867330

[21] Mugwanya KK, Donnell D, Celum C, et al Sexual behaviour of heterosexual men and women receiving antiretroviral pre-exposure prophylaxis for HIV prevention: A longitudinal analysis. Lancet Infect Dis. 2013;13(12):1021–1028. https://doi.org/10.1016/S1473-3099(13)70226-3 24139639 10.1016/S1473-3099(13)70226-3 PMC3920826

[22] Sagaon-Teyssier L, Suzan-Monti M, Demoulin B, et al Uptake of PrEP and condom and sexual risk behavior among MSM during the ANRS IPERGAY trial. AIDS Care. 2016;28(Suppl 1):48–55. https://doi.org/10.1080/09540121.2016.1146653 26883400 10.1080/09540121.2016.1146653 PMC4828609

[23] Martin JN, Roland ME, Neilands TB, et al Use of postexposure prophylaxis against HIV infection following sexual exposure does not lead to increases in high-risk behavior. AIDS. 2004;18(5):787–792. https://doi.org/10.1097/00002030-200403260-00010 15075514 10.1097/00002030-200403260-00010

[24] Cowan FM, Delany-moretlwe S, Sanders EJ, et al PrEP implementation research in Africa: What is new? J Int AIDS Soc. 2016;19(Suppl 6):21101 https://doi.org/10.7448/IAS.19.7.21101 27760680 10.7448/IAS.19.7.21101 PMC5071780

[25] Gill K, Marcus R, Dietrich J, et al An analysis of baseline and early data from the Plus Pills study: An open-label trial of pre-exposure prophylaxis for South African adolescents [homepage on the Internet]. n.d. [2016 Dec 08]. Available from: http://programme.aids2016.org/Abstract/Abstract/10633

[26] MacPhail C, Campbell C ‘I think condoms are good but, aai, I hate those things’: Condom use among adolescents and young people in a Southern African township. Soc Sci Med. 2001;52(11):1613–1627. https://doi.org/10.1016/S0277-9536(00)00272-0 11327136 10.1016/s0277-9536(00)00272-0

[27] Mauck CK, Doncel GF Biomarkers of semen in the vagina: Applications in clinical trials of contraception and prevention of sexually transmitted pathogens including HIV. Contraception. 2007;75:407–419. https://doi.org/10.1016/j.contraception.2007.02.007 17519146 10.1016/j.contraception.2007.02.007

